# The Effect of St. John’s Wort Oil on Urethral Stricture: An Experimental Study in Rat Model*

**DOI:** 10.5152/tud.2025.24068

**Published:** 2025-01-03

**Authors:** Kazım Yelsel, Ergün Alma, Ömer Yüceer, Zeynel Abidin Taş, Berna Totan Ateş, Murat Kobaner, Halil Esimek, Adem Altunkol, Umut Ünal, Ali Ayyıldız

**Affiliations:** 1 Clinic of Urology, Adana Yüreğir State Hospital, Adana, Türkiye; 2 Clinic of Urology, Adana City Training and Research Hospital, Adana, Türkiye; 3 Clinic of Emergency Medicine, Niğde Ömer Halisdemir University, Niğde, Türkiye; 4 Clinic of Pathology, Adana City Training and Research Hospital, Adana, Türkiye; 5 Clinic of Pathology, Adana Yüreğir State Hospital, Adana, Türkiye; 6 Erdemli Alata Horticultural Research Institute, Mersin, Türkiye; 7 Department of Urology, Adıyaman University Faculty of Medicine, Adıyaman, Türkiye

**Keywords:** Urethra,, mucosal inflammation,, St. John’s wort oil,, urethral stricture,, spongiofibrosis

## Abstract

**Objective:**

The aim was to evaluate the effect of yellow and red centaury St. John’s Wort (St. JW) oil on inflammation and urethral fibrosis development in a rat urethral trauma model.

**Methods:**

A total of 24 male rats were divided into 5 groups. No urethral damage was induced in the sham group. The urethras of all rats in the other groups was traumatized at 6 o’clock with a specially designed urethrotome blade. Group 1 was administered 0.5 cc of St. JW oil once daily for 15 days. Group 2 was administered 0.5 cc of red centaury oil intraurethrally once daily for 15 days. The traumatized rats in Group 3 were not treated (St. JW and red centaury oil were not administered). St. JW oil was administered to 3 rats in the Sham 1 group (Group 4), and red centaury oil was administered to 3 rats in the Sham 2 group (Group 5). A 20-G/32-mm intravenous cannula sheath was used for intraurethral administration. On the 15th day, rats were sacrificed and penectomy was performed for histologic evaluation.

**Results:**

Spongiofibrosis, mucosal inflammation, and congestion were significantly decreased in the yellow St. JW oil group when compared with the red centaury oil group and the untreated group (group to which neither St. JW nor red centaury oil was administered).

**Conclusion:**

In this study, intraurethral administration of yellow St. JW oil was found to significantly reduce mucosal inflammation, spongiofibrosis, and obstruction after urethral trauma. According to the results of this study, we think that St. JW oil may be a promising option in the treatment of urethral stricture.

**Main Points**

- Urethral stricture is a common condition characterized by narrowing of the urethra, primarily caused by iatrogenic factors, idiopathic origins, infections, or trauma.
- Hypericum perforatum plant (St.JW) and Hypericum capitatum (H. capitatum) are widely grown worldwide. It is known that yellow and red centaury oil, of which many species also grown in Turkey, has antimicrobial and anti-inflammatory activities and is therefore used in the treatment of wounds and burns.
- St.JW oil was found to show significant results in short-term tissue healing parameters in the rat model after urethral trauma and in this sense, it was considered as an alternative treatment for urethral stricture.

## Introduction

Urethral stricture is a common and sometimes difficult-to-treat disease in urology. Urethral stricture is quite common and is characterized by narrowing of the urethral lumen. It may occur mainly due to iatrogenic and idiopathic causes.^1^ Although less common, it can also be caused by infection and trauma. Fibrosis of the urethra can lead to urethral narrowing, resulting in urethral stricture and voiding dysfunction. If left untreated, many complications may occur. Fibrosis at tissue and cellular levels is a wound repair process and is a pathological process characterized by inflammatory reactions and abnormal activation of myofibroblasts.^2^ Healing and fibrosis due to damage to the corpus spongiosum, a structure surrounding the urethra, or the urethral epithelium cause urethral stricture.^3,4,5^

There are various surgical procedures for the treatment of this disease. However, there is not yet a superior treatment modality for urethral stricture. The variety of treatment modalities reflects the lack of an optimal technique.^6^

St. JW, scientifically known as *Hypericum perforatum* (*H. perforatum*), is a flowering plant used in alternative medicine. Likewise, St. John’s wort (St. JW) oil (*Hypericum capitatum*), used in this study, is also used in many diseases. Since it is believed to be beneficial in many diseases, it has been used for centuries by people in the treatment of various diseases. *H. perforatum* plant (St. JW) and *H. capitatum* are widely grown worldwide. It is known that yellow and red centaury oil, which many species also grown in Türkiye, has antimicrobial and anti-inflammatory activities and is therefore used in the treatment of wounds and burns.^7^

The aim of this study is to demonstrate the effects of St. JW, which has been shown to be effective in wound healing and remodeling and has no serious side effects in the literature so far, on healing, inflammation, and fibrosis development in a urethral injury model and to provide a basis for future studies. For this purpose, St. JW and red centaury oil were used intraurethrally.

## Material and Methods

This study was conducted between September 7, 2022 and September 23, 2022 after approval from the Ethics Committee of Çukurova University Health Sciences Experimental Application and Research Center (ÇÜ-SABİDAM) (03.06.2022/2).

**Figure 1.**
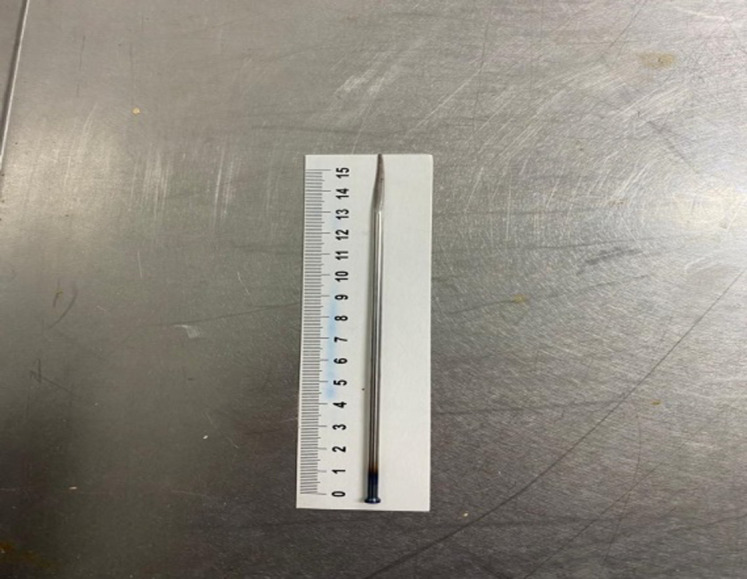
Specially manufactured urethrotome blade.

**Figure 2.**
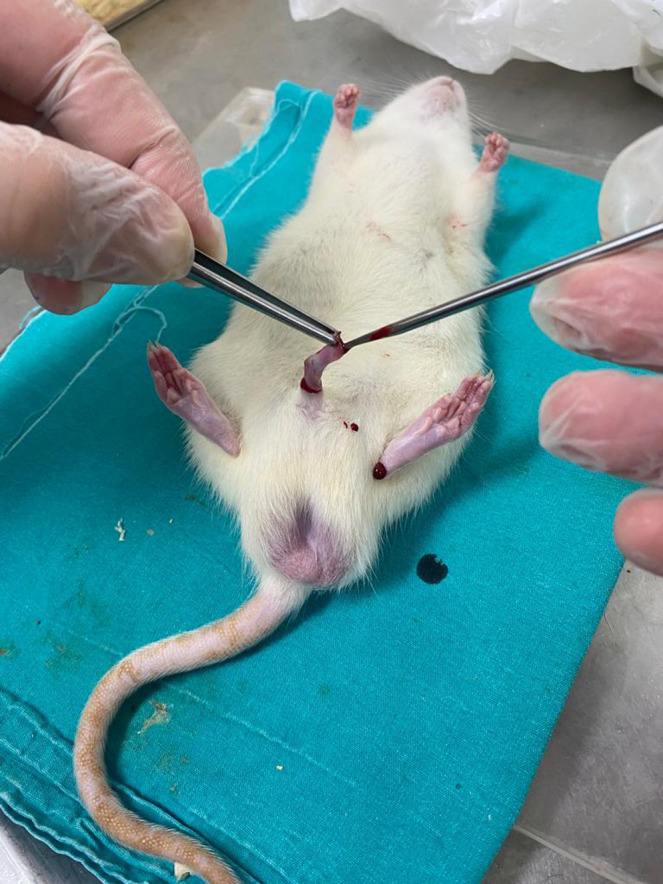
Urethral incision.

A total of 24 Wistar male albino rats weighing between 250 and 300 g were used in the study. Rats were kept in separate cages at room temperature (22°C) with 50% humidity during the preoperative and postoperative periods. On the day of the procedure, the rats were anesthetized with ketamine (50 mg/kg) under sterile conditions. Urethral damage was induced using a urethrotomy blade (tip 3 F, eye 9 F) (Figure 1). A 5-mm longitudinal incision was made 5 mm proximally at the 6 o’clock position, 5 mm proximal to the urethra including the surrounding muscles and the corpus spongiosum. The incision was made in such a way that urethrorrhagia was observed (Figure 2).

**Figure 3.**
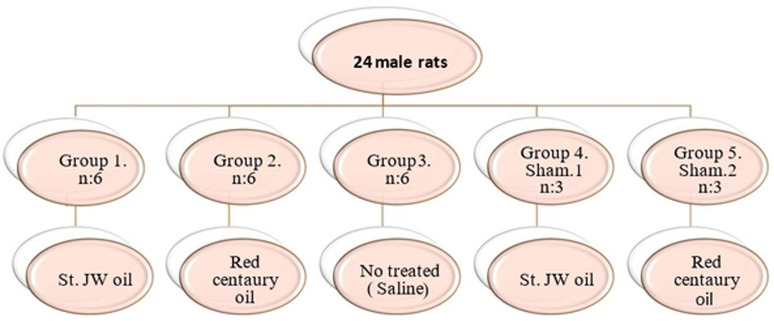
Flowchart of the study.

A total of 24 male rats were divided into 5 groups (Figure 3). No urethral damage was induced in the Sham group. The urethras of all rats in the other groups were traumatized at 6:00 am with a specially produced urethrotome blade. Yellow and red centaury oil was administered intraurethrally once a day to Group 1 (n = 6) and Group 2 (n = 6), respectively, and no treatment was administered to Group 3 (n = 6). Three rats in Sham 1 group received yellow St. JW oil (Group 4), 3 rats in Sham 2 group received red centaury oil intraurethrally (Group 5). A 20-G/32-mm intravenous cannula sheath was used for intraurethral administration. On the 15th day, rats were sacrificed and penectomy was performed for histologic evaluation. Rat penises were placed in 10% formaldehyde and sent to the pathology department for histopathologic examination.

### Preparation of St. JW

Freshly harvested St. JW and red centaury oil plants, with their stems in separate jars, were filled into a wide-mouth glass jar (5 L) containing 350 g, and extra virgin olive oil (approximately 4650 mL) was added to completely cover the plants and not to let air entry (about 4650 mL to fill the jar completely). The lid was tightly closed (0.5 cc of the mixture contains approximately 37.6 mg of extract). The jar was kept in sunlight for 45 days, then filtered and filled into bottles.

Red centaury oil is derived from the same plant species as St. JW oil. The difference is that red centaury oil is obtained from fresh plants, while St. JW oil is obtained from dried plants.

### Histopathological Analysis

Histopathologic analysis was performed by an independent pathologist under a light microscope. The specimens (urethral tissues) were fixed in 10% formalin in a separate container for each rat until the time of histologic examination. These tissue samples were cut into squares 3 mm apart and embedded in paraffin blocks. For histochemical examination, 4-micron thick sections were taken from the paraffin blocks and stained with hematoxylin & eosin and Masson trichrome. The preparations were examined under a light microscope at 200× magnification. In the histopathologic examination of the tissues, spongiofibrosis, inflammation, congestion, and calcification/metaplasia were evaluated (Table 1).

### Statistical Analysis

First, we analyzed the measurements to determine the sample size for the study. The effect size was calculated based on the level of fibrosis using power analysis. The exact value was adjusted to *d* = 0.80. As a result of the analysis, 3 rats were required for the Sham group to achieve an accurate result with a 5% chance of error and a 95% chance of obtaining the power value. Since 24 rats were recruited for the study, the study was completed with this sample size.

The data were analyzed using the statistical software SPSS v.20.0 statistical software (IBM SPSS Corp.; Armonk, NY, USA). The values taken during the first examination were used to perform a power analysis. The *χ*² test was used to determine if there were distinctions among categorical variables distributed in different groups. Categorical variables were calculated as frequency and percentage scores, and results of the statistical analysis were assumed to be significant if the *P* value were < .05.

## Results

**Figure 4.**
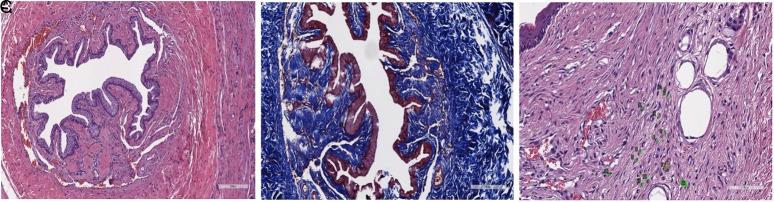
(A) Congested vascular structures and fibrosis in the connective tissue under the urothelial epithelium (hematoxylin & eosin x200). (B) In the same specimen, increased fibrosis is seen with more intense staining in Masson trichrome staining (Masson trichrome x200). (C) Pigmented hemosiderin-laden macrophages among the connective tissue under the urothelial epithelium (hematoxylin & eosin x200, some of the macrophages are marked and numbered).

Spongiofibrosis, inflammation, congestion, and calcification/metaplasia were evaluated in the histopathological examination of tissues of all rats (Figure 4). No death was detected in rats during or after the study.

Group analysis comparing groups 1, 2, 3, 4, and 5 showed a significant difference in spongiofibrosis, mucosal inflammation, and congestion (*P* < .001, <.001, <.001, <.002, respectively). No statistically significant difference was observed between the groups in calcification/metaplastic scoring (*P* = .217) (Table 2). Spongiofibrosis, hemosiderin, and congestion were statistically different between the groups. This difference was due to the difference between Group 1, Group 2, and Group 3. Histologic scoring showed that Group 1 (St. JW oil treated group), Group 4 (Sham 1), and Group 5 (Sham 2) had the lowest scores for spongiofibrosis, mucosal inflammation, and congestion. Group 2 (red centaury oil) and Group 3 (no treatment) had higher spongiofibrosis, mucosal inflammation, and congestion scores than Group 1 (St. JW) (*P* < .001, <.001, <.002, respectively).

**Figure 5.**
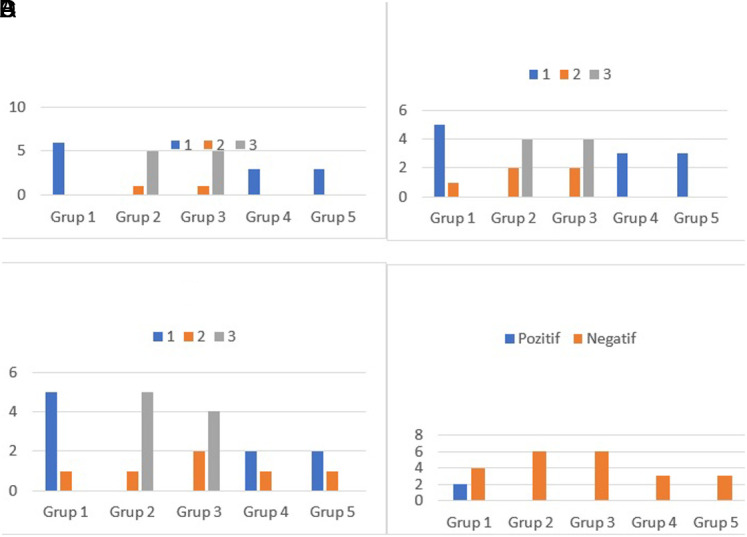
Differences between the groups. (A) Level of spongiofibrosis in groups (*P* < .001), (B) Level of hemosiderin-laden macrophages in groups (*P* < .001), (C) Level of congestion in groups (*P* < .001), (D) Calcification/metaplasia positivity in groups (*P* < .002).

Spongiofibrosis, mucosal inflammation, and congestion were significantly different in the St. JW-treated group compared to the red centaury oil-treated and untreated groups (*P* < .001, <.001, <.001, <.002, respectively). Spongiofibrosis, mucosal inflammation, and congestion scores were similar between Group 1, Group 4, and Group 5 (Figure 5).

## Discussion

It was aimed to evaluate the effect of yellow and red centaury (St. JW) oil on inflammation and urethral fibrosis development in a rat urethral trauma model. In this study, intraurethral administration of yellow St. JW oil was found to significantly reduce mucosal inflammation, spongiofibrosis, and obstruction after urethral trauma. 

Urethral stricture is characterized by healing and fibrosis responses (spongiofibrosis) of the urethral mucosa and surrounding spongiosis tissues in male patients. Fibrosis is a pathological wound repair process and is characterized by abnormal proliferation of myofibroblasts at the cellular level, resulting in urethral stricture. Urethral strictures may develop due to many causes (idiopathic, iatrogenic, infection, and trauma).^4^ Idiopathic causes are more common in young people and may occur due to developmental anomalies or traumas. In the elderly, it may develop due to decreased blood flow. Iatrogenic causes are usually associated with surgery or catheterization. The causes of infection have decreased with the increased use of antibiotics. Urethral strictures caused by trauma may occur due to various accidents, especially traffic accidents and falls. Some surgical interventions can also cause urethral strictures. If urethral strictures are not treated in time, some complications may occur. These complications may range from urinary tract infection to bladder and kidney damage.^2,3,4,5^

After the development of urethral stricture, long-term endoscopic interventions, urethral dilatation, and/or intermittent catheterization procedures may be necessary. In surgical procedures in which open surgery may be necessary, end-to-end anastomoses and/or free grafts may be required.^8^ However, the overall recurrence rate for reconstructive procedures is quite high, although it varies depending on the location and type of stenosis. Currently, there is still no effective treatment to prevent the formation of fibrosis, increase the success rate of surgical treatments, and decrease the recurrence rates.^9^ Antifibrotic drugs such as somatostatin analogs, glucocorticoids, and platelet rich plasma (PRP) have been used to prevent urethral stricture after surgery but have not been included in treatment protocols.^10-17^

Today, with the development of both optical systems and endoscopic instruments, the use of endoscopy in urologic procedures has increased, and the possibility of iatrogenic urethral injury has increased accordingly.^18^ Especially the use of endoscopic instruments with inappropriate urethral diameters is one of the factors that cause these injuries.^19^ Some studies have shown that most urethral strictures are the result of non-diathermic iatrogenic urethral trauma.^1^ In our prospective study, a non-diathermic iatrogenic urethral trauma model was developed.

St. Johns Wort grows almost everywhere in the world, especially in Europe, Africa, and certain regions of Asia, with the most endemic species growing in Anatolia. It has been used as a healing herb in many civilizations for centuries, since the ancient Greeks and Romans. It has also been traditionally used in wound healing for centuries, and its effectiveness has been demonstrated in many publications. St. John’s Wort and red centaury are still widely used today, both as oils and teas. The range of uses in disease is quite wide. These include many different areas such as insomnia, kidney disorders, gallbladder disorders, and liver disorders.^20^

In Türkiye, St. JW obtained from dried plants and red centaury oil obtained from fresh plants have healing effects due to the various substances in their content. Especially, St. JW has been shown to have an effect on wound healing in literatüre.^21^ This is provided by some substances in its structure. Although there are many compounds in its structure, the most active ones are hyperforin (fluoroglucinol derivative) and hypericin (naphthodiantrone derivative). Hyperforin is responsible for various therapeutic effects of the plant, such as antibacterial, anti-inflammatory, antidepressant, antitumoral, and antioxidant effects.^22,23,24,25^ One of the characteristic compounds of St. JW is hypericidin. Hypericidin is responsible for antimicrobial, antiviral, and anti-inflammatory effects. In addition, quercetin, which is involved in the inhibition of monoamine oxidase A, reduces free radical formation in tissues and contributes to wound healing. St. JW also contains flavonoids such as kaempferol, biapigenin, and amentoflavone, which also contribute to wound healing.^26^

Kafadar et al^27^ showed that St. JW was effective in wound healing in their study. The expression levels of collagen III, proinflammatory (TNF-α and IL-6), and anti-inflammatory (TGF-ß1) factors were compared in the wound healing model, and it was found that collagen III and TGF-ß1 were less concentrated, while IL-6 and TNF-α were more concentrated in the wound area. Similarly, fibrosis, inflammation, and congestion were found to be lower in the St. JW group compared to the other groups in our study, suggesting that St. JW can be used for the treatment of urethral stricture.

St. JW has been used for years in the local treatment of many diseases, including burns, due to these antifibrotic effects. Saljic et al used St. JW for wound healing in burns and found that wound healing was accelerated and scar formation was decreased compared to the non-used group.^28^ Samadi et al^29^ also showed that St. JW accelerated the wound healing and decreased scar formation after cesarean section. Yücel et al^30^ reported that St. JW can be used effectively in the treatment of pressure sores in intensive care patients. Guvenc et al^31^ investigated the efficacy of St. JW in corrosive esophageal burns in rats and found that St. JW reduced stenosis. In our study, we focused on urethral strictures, a condition in which St. JW has not been used before and recurrences are frequently observed after endoscopic treatment. The known antifibrotic effects of St. JW were demonstrated in our study similarly to these previous studies, and it was aimed to guide other similar studies to be conducted in the future.

Our study has some limitations. The most important limitation of this study is its short follow-up period and the limited number of subjects. Long-term results are important in the treatment of urethral stricture since it is a chronic disease. This study reflects short-term results in an animal model. To our knowledge, there is no other study in which St. JW was used as a treatment option after urethral injury, and this study is considered valuable in this sense.

St. JW oil was found to show significant results in short-term tissue healing parameters in the rat model after urethral trauma and in this sense, it was considered an alternative treatment for urethral stricture. However, this needs to be supported by further large-scale and long-term studies.

## Figures and Tables

**Table 1.** Histopathologic Evaluation Parameters

Spongiofibrosis
0 None
1+ <10% tissues with fibrosis
2+ 10%-49% tissues with fibrosis
3+ >50% tissues with fibrosis
Inflammation
0 None
1+ <5 Hemosiderin-laden macrophages/×200 magnification
2+ 5-10 Hemosiderin-laden macrophages/×200 magnification
3+ 10< Hemosiderin-laden macrophages/×200 magnification
Congestion
0 None
1+ Mildly severe
2+ Moderately severe
3+ Severe
Calcification/metaplasia
0 No
+ Yes

**Table 2.** Pathological Data and *P* Values Between Groups

	Groups					*P*
	1	2	3	4	5	
Spongiofibrosis						
1	6 (100%)	0 (0%)	0 (0%)	3 (100%)	3 (100%)	**<.001**
2	0 (0%)	1 (17%)	1 (17%)	0 (0%)	0 (0%)	
3	0 (0%)	5 (83%)	5 (83%)	0 (0%)	0 (0%)	
Hemosiderin						
1	5 (83%)	0 (0%)	0 (0%)	3 (100%)	3 (100%)	**<.001**
2	1 (17%)	2 (33%)	2 (33%)	0 (0%)	0 (0%)	
3	0 (0%)	4 (67%)	4 (67%)	0 (0%)	0 (0%)	
Congestion						
1	5 (83%)	0 (0%)	0 (0%)	2 (67%)	2 (67%)	**.002**
2	1 (17%)	1 (17%)	2 (33%)	1 (33%)	1 (33%)	
3	0 (0%)	5 (83%)	4 (67%)	0 (0%)	0 (0%)	
Calcification						
Negative	2 (33%)	0 (0%)	0 (0%)	0 (0%)	0 (0%)	.217
Positive	4 (67%)	6 (100%)	6 (100%)	3 (100%)	3 (100%)	

## Data Availability

The data of this study is available upon request to the corresponding author.
